# Case Report: Perhaps we can do more when paradoxical embolism meets thrombophilia: inspiration from a special case

**DOI:** 10.3389/fcvm.2025.1608644

**Published:** 2025-08-12

**Authors:** Xiaomin Xue, Muhua Dai, Lisha Pang, Jiayan Li, Chunlian Ji, Jianbiao Meng, Wei Zhang

**Affiliations:** ^1^ Institute of Basic Experiments, Zhejiang Academy of Traditional Chinese Medicine, Hangzhou, China; ^2^ Department of Critical Care Units, Tongde Hospital of Zhejiang Province, Hangzhou, China; ^3^ Department of Cardiology, Tongde Hospital of Zhejiang Province, Hangzhou, China

**Keywords:** paradoxical embolism, pulmonary embolism, acute cerebral infarction, venous thromboembolism, plasminogen activator inhibitor-1

## Abstract

This study reported a rare case of Paradoxical Embolism complicated with thrombophilia. New thrombosis still existed after active treatment, and the existence of thrombus-prone gene was confirmed by gene detection. This patient recovered after improving the treatment plan. It emphasizes the importance of genetic testing to improve the diagnosis and treatment plan. This study comprehensively recorded the whole diagnosis and treatment process, including multidisciplinary collaboration, imaging diagnosis and personalized treatment strategies, which provided valuable guidance for similar clinical cases.

## Introduction

Thromboembolic diseases, including myocardial infarction, ischemic stroke and pulmonary embolism (PE), are the most common diseases that threaten human life and health (1). Paradoxical Embolism (PDE) is a rare thromboembolic disease with a high mortality rate, which can reach 62.5% within the first 24 h (2). PDE is characterized by thromboembolism, which originates from the right heart or venous vascular system and forms arterial embolism through right-to-left shunt (RLS) channel (3). Venous thromboembolism (VTE) includes PE and deep venous thrombosis. Approximately 50% of VTE has at least one hereditary or acquired thrombophilia (4). Thrombophilia is defined as abnormal susceptibility to thrombosis, which has genetic susceptibility to lead to hypercoagulability and increase the risk of thromboembolic events (5, 6). As a rare case, PE can lead to PDE. Although similar reports have been reported, there is an absence of both thrombophilia and thrombophilia gene detection (7, 8). Genetic testing has important reference value in making treatment plans, which can improve treatment and reduce the risk of recurrence (9).

Different from previous reports, this study reported a rare case of PDE complicated with thrombophilia. A 70-year-old female patient suffered from PE complicated with acute cerebral infarction. New thrombosis still existed through active thrombolysis, interventional embolectomy and anticoagulant therapy. Genetic testing confirmed the existence of thrombogenic genes. Follow-up to improve the treatment plan, and long-term follow-up, the patient's condition was successfully controlled, and no thromboembolism occurred again.

## Patient information

A 70-year-old female was admitted to the hospital at 16: 00 on December 13, 2021, because of “chest tightness with shortness of breath for more than 10 days”. The patient suffered from experienced chest tightness and shortness of breath for over 10 days, without obvious triggers, which can be relieved after a few minutes' rest, with occasional cough, no fever, no chest pain, no nausea and vomiting, no palpitation and sweating, no dizziness and other discomfort. These symptoms are usually relieved after a few minutes' rest. She didn't take it seriously and the symptoms flared up and recurred. Symptoms often occur obviously after activities, and chest tightness and shortness of breath are obvious after climbing the second floor. The patient had a history of “hypertension”, which was controlled by oral antihypertensive drugs. The patient exhibited no additional medical history. Admitted physical examination yielded no noteworthy findings.

**Figure 1:**
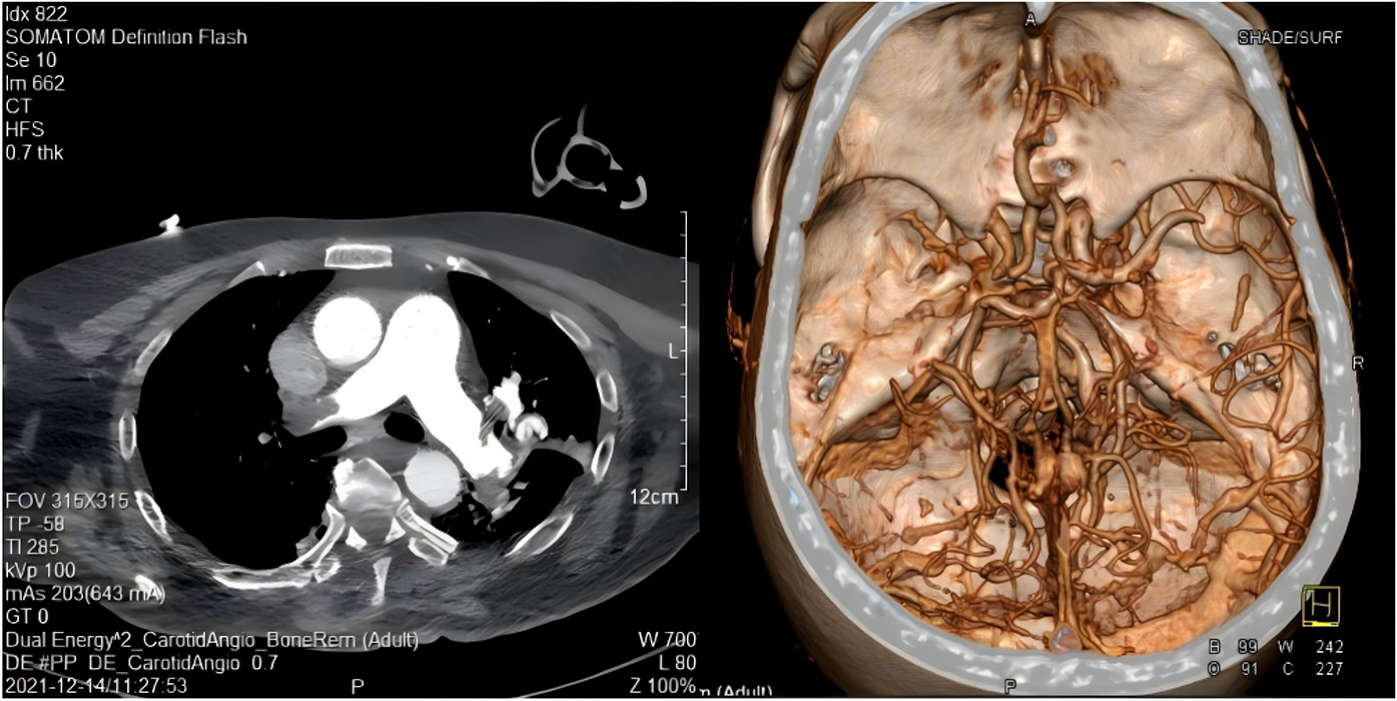
Cervical CTA on December 14, 2021.

At 10:00 am on December 14, the patient's condition suddenly deteriorated. She experienced syncope accompanied by limb shaking, deep breathing with snoring, and decreased oxygenation, with the lowest oxygen saturation recorded at 78%. A physical examination at that time revealed the following: blood pressure 161/85 mmHg, heart rate 100 beats per minute, bilateral pupils equal in size (approximately 2 mm in diameter) with intact light reflex, and bilateral Babinski sign positive. Blood gas analysis showed a partial oxygen pressure of 45 mmHg. Further investigations were conducted to determine the underlying cause. A head CT scan ruled out cerebral hemorrhage. However, the patient's altered consciousness could not exclude the possibility of an acute stroke. A computed tomography angiography (CTA) of cervical vessels was performed immediately, which revealed left middle cerebral artery occlusion and bilateral pulmonary embolism (Figure 1) (Attachment 1). The color Doppler ultrasound of blood vessels in both lower limbs on the same day showed multiple small plaques in the arteries of both legs and blood stasis in the posterior tibial vein of the left lower limb (Attachment 2).

Pulmonary embolism severity index (PESI), an important tool to evaluate the severity and prognosis of patients with PE, can effectively guide clinical risk stratification and prognosis judgment (10). According to PESI scoring system, Grade III and above (Grade III-V) are defined as high risk of mortality, which is associated with increased PE-related mortality, and serious adverse events (11). The patient was classified as PESI grade IV, suggesting that active intervention was urgently needed. After joint consultation with multidisciplinary teams of cardiovascular medicine, respiratory medicine and neurology, it was finally decided to implement emergency thrombolytic therapy (alteplase scheme) combined with catheter interventional embolectomy to address the acute conditions.

**Figure 2:**
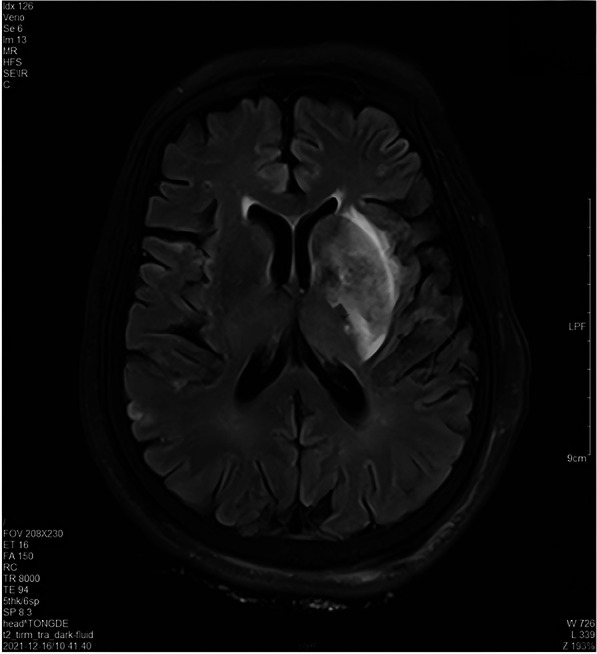
Cranial MRI on December 16, 2021.

The brain MRI on December 16th showed acute multiple cerebral infarction (Figure 2) (Attachment 3). Concurrently, a right echocardiogram confirmed the presence of RLS, suggesting the existence of patent foramen ovale or an atrial septal defect (Attachment 4). The diagnosis of PDE must meet three criteria: VTE, intracardiac shunt or pulmonary fistula and arterial embolism (12). The diagnosis of PDE is clear based on the above imaging evidence. After the contraindications were eliminated, rivaroxaban was taken orally for anticoagulant therapy, with the dosage of rivaroxaban being adjusted according to renal function. Notwithstanding prior administration of thrombolysis and oral anticoagulation therapy, coagulation function testing revealed persistent augmentation in D-dimer levels and persistent reduction in fibrinogen levels. Concurrently, the ultrasound examination of the lower extremity vasculature revealed the occurrence of new venous thrombosis (Attachment 5). There are many potential risk factors for thrombosis. We perfected the anti-neutrophil antibody combination + antinuclear antibody series (all), immunoglobulin A/G/M, anticardiolipin antibody, anti-β2 glycoprotein and other tests during the treatment, and all of them were negative. Based on the analysis of the diagnosis and treatment standards for VTE, the medical team believed that the patient may be complicated with thrombophilia. Hence, we added the detection of thrombophilia gene. In this paper, matrix-assisted laser desorption ionization time-of-flight mass spectrometry (MALDI-TOF) was used to detect 9 thrombogenic genes (PROC, PROS1, SERPINC1, F2, F5, HRG, THBD, PAI-1, MTHFR) and 27 hot spot variations. The detection of thrombus-prone genes revealed a thrombophilic risk factor: plasminogen activator inhibitor-1 (PAI-1) (4G/4G) (Attachment 6).

Through active rescue treatment, the patient's pulmonary thrombus load gradually decreased, the oxygenation index improved obviously, exceeding 300mmHg, and the percutaneous oxygen saturation was maintained at 98%–100%. Ultrasound examination showed that the right ventricular function improved rapidly, the right ventricle gradually returned to normal size, the pulmonary artery pressure continued to drop, and the condition gradually improved. With the gradual improvement of symptoms of cerebral infarction, the consciousness was obviously improved, and the muscle strength of upper and lower limbs was significantly increased. She recovered and was discharged on January 15th, 2022.

After discharge, we have been given long-term adequate anticoagulant therapy and long-term life guidance in combination with the patient's thrombophilia. Post-discharge follow-up continued until May 2023. During this period, the patient did not experience thromboembolic events.

## Discussion

As a product of fibrin formation and degradation, D-dimer reflects the activation state of coagulation system and fibrinolysis system *in vivo* and is widely used in the diagnosis of VTE (13). Fibrinogen plays many roles in thrombosis, including stimulating platelet aggregation and increasing blood viscosity (14). The continuous increase of D-dimer and the decrease of fibrinogen during hospitalization indicated that the coagulation system and fibrinolysis system were continuously activated, and the risk of thrombosis was increased. Those findings were consistent with vascular ultrasound examination, which confirmed the persistence of thrombosis and dissolution.

The high expression of PAI-1 usually leads to the dysfunction of coagulation and fibrinolysis system, which leads to thrombosis (15). Fibrinolytic system is responsible for dissolving thrombus and preventing thrombosis, and PAI-1 is the main inhibitor of fibrinolytic system (16). PAI-1 is a serine protease inhibitor, which reduces the production of active plasmin by inhibiting tissue plasminogen activator. Plasma PAI-1 concentration in humans with PAI-1 4 g/4G genotype increases, which leads to the damage of fibrinolytic system, and promotes the increase of coagulation and the formation of microthrombosis (5, 15, 17).

With active anticoagulation therapy, the patient still forms a new thrombus, so we will naturally have questions: insufficient anticoagulation? Or is the risk factor of thrombosis too strong? Or is the risk factor of thrombosis too strong? This question had been perfectly explained through the report of genetic testing.

Two special diseases meet unexpectedly in real cases when PDE combined with thrombophilia. As a special and rare case, this study may be the first report in academic circles. The treatment plan should not be doubted because of thrombosis secondary formation. We should doubt whether there are some influencing factors that make thrombosis easier to form, such as thrombophilia. The case emphasizes the close relationship between thrombophilia gene, thrombosis and thromboembolic diseases, and provides strategies and evidence for the treatment and prevention of thromboembolic diseases.

## Conclusions

Generally speaking, VTE can lead to serious complications, such as PE and PDE. Clinicians should consider the possibility of thrombophilia when facing VTE, especially in patients with repeated thrombosis or secondary embolism. Clinical workers should strengthen the collection of medical history, and some patients can highly doubt the existence of thrombophilia through medical history collection. To cases with recurrent thrombosis or secondary embolism, the addition of gene screening is beneficial to make more accurate and effective treatment plans, make more targeted prevention guidance, improve prognosis and reduce the risk of recurrence.

## Data Availability

The original contributions presented in the study are included in the article/Supplementary Material, further inquiries can be directed to the corresponding author.

## Supplementary material

Supplementary Presentation 1 Graphical abstract.

Supplementary Figure 1 Report of cardiac Doppler ultrasound examination.

Supplementary Video 1 The image of right heart contrast echocardiography.
