# Supplementary figures and images for: Case Report: Perhaps we can do more when paradoxical embolism meets thrombophilia: inspiration from a special case
Source: Front Cardiovasc Med. 2025 Aug 12;12:1608644. doi: 10.3389/fcvm.2025.1608644 (PMC12379029; doi:10.3389/fcvm.2025.1608644)

## Slide 1
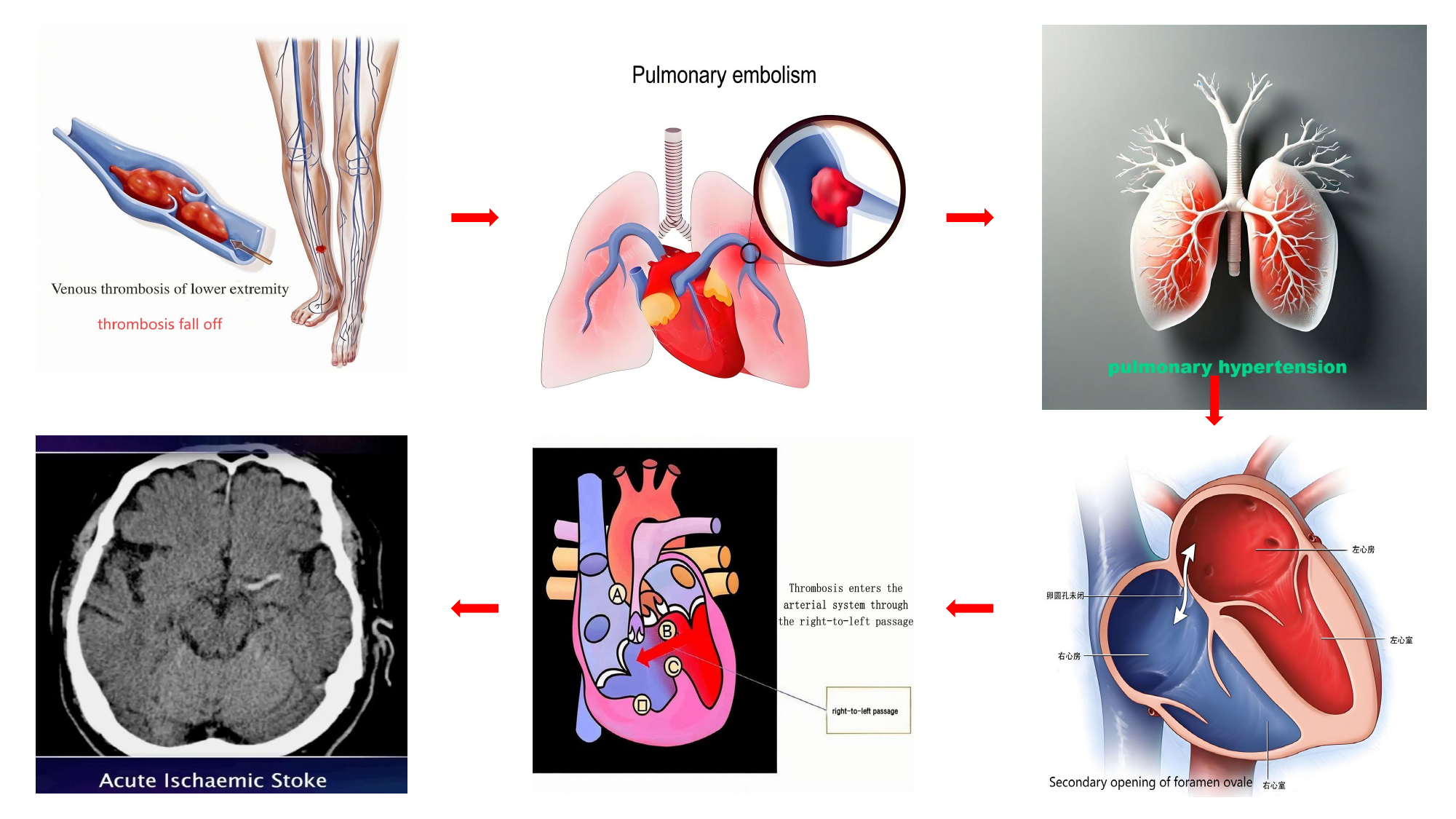

Supplement: Supplementary Presentation 1 [file Presentation1.pptx]

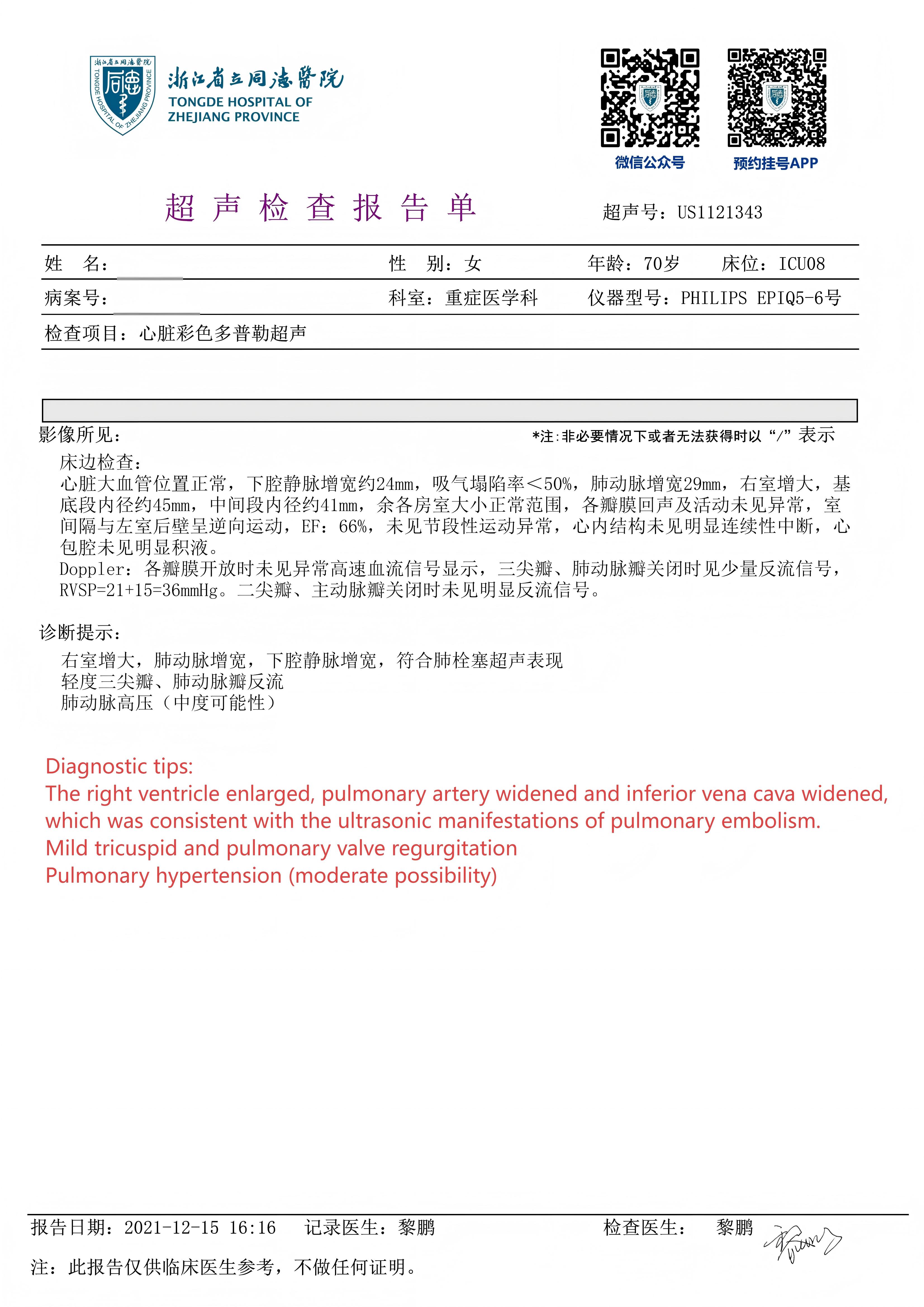

Supplement: Supplementary Figure 1 [file Image1.jpg]
